# Dhat Syndrome Presenting as Secondary Depression in a Patient With Mild Intellectual Disability

**DOI:** 10.7759/cureus.66103

**Published:** 2024-08-03

**Authors:** Abinaya Kalimuthu, Aruna Kaki, Ramya Rachel Jetty, Madhusudan T, Arul Saravanan R

**Affiliations:** 1 Psychiatry, SRM Medical College Hospital and Research Centre, Kattankulathur, IND

**Keywords:** culture-bound syndrome, cognitive behavioral therapy, intellectual disability, depression, dhat

## Abstract

Dhat syndrome is a condition in which the patient is morbidly preoccupied with excess loss of semen either from urination, nocturnal emissions, or masturbation. The anxiety surrounding this is based on the cultural and societal belief that excessive semen loss will result in illness. Here, we report a case of a 34-year-old male with intellectual disability who presented with depressive symptoms and on detailed exploration was found to have Dhat syndrome. The challenging part in this case was eliciting psychopathology and giving psychotherapy as the patient had mild intellectual disability. We treated this patient successfully with a combination of Manualised Individual Cognitive Behavioural Therapy (M-iCBT) and pharmacotherapy. This case report highlights the importance of exploring sexual history and screening for culture-bound syndromes in patients with mild intellectual disability.

## Introduction

Culture-bound syndromes are conditions in which the psychiatric manifestations are in close association with cultural factors and thus warrant primary management from a cultural perspective. Culture-bound syndromes are now widely identified because of rapid globalization and immigration, which has led to the intermingling of cultures. Researchers described these conditions by local folk names, like susto, Dhat, hwa-byung, and nervios. These conditions are confined to certain cultures and hence the name culture-bound syndromes [1].

The term "Dhat" is derived from the Sanskrit word "Dhatu," which means “metal” or “medicinal constituent,” and is considered a powerful material in the human body. It is more common in men, especially in Southeast Asian countries. Many people believe that Dhat is a bodily humor that begins as food and gets progressively converted to blood, bone marrow, and semen [2]. Hence, people attribute the loss of semen to the loss of Dhat, which is considered a loss of vitality. Anxiety, which surrounds the concept of excessive semen loss, leads to Dhat syndrome. Major depressive disorder is a common diagnosis among Dhat syndrome patients [3].

Sexual health is often overlooked despite its significant impact on mortality, morbidity, and disability. Dhat syndrome is associated with prejudices, myths, and fallacies [4]. Because of the awkwardness and discomfort of discussing this area, patients initially present with symptoms of anxiety and depression. Furthermore, patients with intellectual disability will have difficulty in conceptualizing and verbalizing their problems, which will add to their distress.

## Case presentation

A 34-year-old married male from a lower socioeconomic background, belonging to a Hindu nuclear family, who did not complete his secondary schooling and has poor occupational functioning, hailing from a rural area in Southern India, presented with complaints of generalized fatiguability, decreased sleep, low mood, and suicidal ideation over the past six months. His family members reported that he was confined to his room, not watching TV, not going to work, or not participating in social events. When family members asked about his problems, he expressed his wishes to die but did not tell them why. He would also feel lethargic and frequently complained of body aches and tiredness.

He had a similar presentation along with a history of one suicide attempt four years before the current presentation. His family members also reported that he had frequent altercations with his wife and she has been living separately for the past four years. From the developmental perspective, his academic skills were poor and he could not complete his secondary education as he had difficulty in understanding the subjects. He had much difficulty in performing mathematics. He could not perform simple calculations and had difficulty managing matters involving money. He came to know about masturbation from his school friends. Initially, he was masturbating occasionally, and it has increased over the past four years. He started watching pornography videos with school friends during their holidays, and later he continued to watch the videos alone almost every day. His parents also gave a history of the patient touching his private parts in public in his adolescence, and they had much difficulty in getting him out of the habit. He got married five years ago, and both partners were not satisfied with their sexual lives and have been separated for the past four years.

A thorough physical examination was carried out, and nothing significant was observed. On the initial Mental Status Examination, there was poor eye contact, difficulty in sustaining his attention, answering the questions in one or two words, somatic preoccupation, and dull affect. The patient was admitted to the psychiatry inpatient care, and a diagnosis of recurrent depressive disorder was made initially.

On the Serial Mental Status Examination, the patient reported that he had been experiencing extreme tiredness and vague body pain and was not able to concentrate on his work. He also complained of feeling low and not interested in talking with anyone and expressed his death wishes. He attributed all these symptoms to his excessive involvement in watching pornography and having masturbatory practice since his puberty. His masturbatory practice has increased over the past four years, and this has led to intermittent and repeated loss of milky white, odorless sticky fluid in his urine. The patient could not understand the nature and composition of the fluid and started to worry and remain preoccupied about the same. He came to know from the mass media that any excessive secretion from his genitals would drain his energy and make him impotent. He also reported that he started to avoid having sexual intercourse with his wife as he had a fear of not performing well in the intercourse. He also feared that he would be framed as impotent by his wife and other family members as he had lost an excess amount of white fluid from his genitals. As he was constantly avoiding sexual intercourse with his wife without giving her proper reasons, they had frequent arguments, which led to their separation and increased his distress. The patient also said that he had shared his problem with his friend who had taken him to a faith healer and Ayurveda practitioner, but he was not relieved of his distress.

A sexual dysfunction workup was done to rule out primary sexual dysfunction. When the Hamilton Depression Rating Scale [5] was applied, it revealed a score of 22, which signifies moderate depression. The patient was started on tablet sertraline 50 mg, which is an antidepressant, and it was increased to 150 mg by the end of the first week. His depressive symptoms decreased by the end of the third week. The Hamilton Depression Rating Scale was repeated, and it revealed a score of 12. His affect was euthymic, and he appeared energetic. An intelligent quotient (IQ) assessment was done by using Binet Kamat Test of Intelligence in the fourth week; his score was 65, which signifies a mild deficit in intellectual functioning. We started him on Manualised Individual Cognitive Behavioural Therapy (M-iCBT), which is a personalized cognitive therapy useful in cases of patients with intellectual disability [6]. We gave once-weekly one-hour sessions for 16 weeks. We assessed the patient’s knowledge about sexual health, introduced the concept of semen, and addressed his false beliefs surrounding it. The purpose of this session was to separate his false ideas from reality, put things into perspective, help the patient retain healthy thinking, and finally discard his faulty thinking. The patient was engaged in meaningful employment as a farm helper to ensure adequate occupational functioning.

The patient has been maintaining well with tablet sertraline 150 mg. His socio-occupational functioning improved. He is being compliant with medication and is on regular follow-up.

## Discussion

Dhat syndrome is usually seen in middle-aged males from rural backgrounds [2]. There have been various psychiatric comorbidities associated with Dhat syndrome and the most common is depression. Patients with Dhat syndrome associate their tiredness, low mood, and loss of interest with loss of semen, thereby resulting in their low sexual performance, which is seen in our patient [7]. Many authors do consider Dhat syndrome as a part of the spectrum of depressive disorder because of its overlapping phenomenology with depression [8]. A study done by Chadha to find comorbidities in Dhat syndrome revealed that 50%, 32%, and 18% were related to depression, somatoform disorders, and anxiety, respectively [9].

Adolescents with intellectual disability are engaged in several sexual behaviors, and masturbation is the most commonly found solitary behavior, while others are touching genitals and getting naked in public [10]. Our patient’s parents also reported the patient’s habit of touching his genitals in public places during his adolescent period. Jahoda and Pownall in their study found that younger people with intellectual disability have less sexual knowledge when compared to their peers from the general population even in a community with accentuated sex education programs [11]. McCabe in his study noted that people with intellectual disability were less likely to discuss their sexual problems with their family members or friends, which will lead to a lack of normalization of sexual health-related problems and limit their sexual knowledge [12].

A study conducted by Prakash et al. has revealed a gross disparity in understanding the concept of Dhat syndrome and also its management by alternate medical systems like homeopathy, Ayurveda, Siddha, Unani, and faith healers [13,14]. Because of this disparity, patients get confused, and they often visit multiple healthcare centers [15]. This is the same scenario in our patient who previously visited a faith healer and Ayurveda practitioner before seeking help from the psychiatrist.

A study conducted by McCabe et al. on 34 patients with intellectual disability showed that there was a significant improvement in terms of depression in patients who underwent cognitive behavioral therapy compared to the control group [16]. A pilot study conducted by Hartley showed that adults with mild intellectual disability benefitted from a 10-week group cognitive behavioral therapy [17]. A meta-analysis conducted by Graser and his colleagues has shown the efficacy of cognitive behavioral therapy in the management of anger and depressive symptoms in patients with intellectual disability [18]. Azam et al. in 2012 described a treatment manual based on a transdiagnostic perspective on anxiety and depression [19]. Apart from treating depression and anxiety in patients with intellectual disability, studies have shown the effectiveness of M-iCBT in anger management in these patients [20].

Our patient who had lesser knowledge about masturbation was misguided under the influence of mass media, which caused distress in him. Because of his intellectual disability, he had difficulty verbalizing his problem, which further escalated his distress and led to the development of Dhat syndrome. In our patient with mild intellectual disability, we not only identified Dhat syndrome but have also successfully treated him with a combination of M-iCBT and pharmacotherapy. This case report highlights the importance of exploring sexual history and screening for culture-bound syndromes in patients with mild intellectual disability.

## Conclusions

Culture-bound syndromes, like Dhat syndrome, can be challenging to diagnose due to the stigma surrounding sexual health and the varied treatment options available from different medical systems. Integrating these varied medical systems is necessary to benefit the patient and alleviate their suffering as quickly as possible. There is a risk of mass media misguiding society, making sex education essential for imparting appropriate knowledge. Diagnosing conditions associated with sexual health is even more difficult in individuals with intellectual disabilities. Therefore, a detailed psychosexual development history, physical examination, and laboratory investigations are necessary to identify both primary sexual dysfunction and psychiatric conditions. The literature supports the effectiveness of M-iCBT in patients with mild to moderate intellectual disability. Consequently, M-iCBT, in conjunction with pharmacotherapy, can effectively benefit these patients when necessary.
